# Can Facebook Be Used for Research? Experiences Using Facebook to Recruit Pregnant Women for a Randomized Controlled Trial

**DOI:** 10.2196/jmir.6404

**Published:** 2016-09-21

**Authors:** Laura M Adam, Donna P Manca, Rhonda C Bell

**Affiliations:** ^1^ Department of Agricultural, Food & Nutritional Science Faculty of Agricultural, Life & Environmental Sciences University of Alberta Edmonton, AB Canada; ^2^ Department of Family Medicine Faculty of Medicine & Dentistry University of Alberta Edmonton, AB Canada

**Keywords:** pregnant women, maternal health, social media, Internet

## Abstract

**Background:**

Recruitment is often a difficult and costly part of any human research study. Social media and other emerging means of mass communication hold promise as means to complement traditional strategies used for recruiting participants because they can reach a large number of people in a short amount of time. With the ability to target a specified audience, paid Facebook advertisements have potential to reach future research participants of a specific demographic. This paper describes the experiences of a randomized controlled trial in Edmonton, Alberta, attempting to recruit healthy pregnant women between 8 and 20 weeks’ gestation for participation in a prenatal study. Various traditional recruitment approaches, in addition to paid Facebook advertisements were trialed.

**Objective:**

To evaluate the effectiveness of paid advertisements on Facebook as a platform for recruiting pregnant women to a randomized controlled trial in comparison with traditional recruitment approaches.

**Methods:**

Recruitment using traditional approaches occurred for 7 months, whereas Facebook advertisements ran for a total of 26 days. Interested women were prompted to contact the study staff for a screening call to determine study eligibility. Costs associated with each recruitment approach were recorded and used to calculate the cost to recruit eligible participants. Performance of Facebook advertisements was monitored using Facebook Ads Manager.

**Results:**

Of the 115 women included, 39.1% (n=45) of the women who contacted study staff heard about the study through Facebook, whereas 60.9% (n=70) of them heard about it through traditional recruitment approaches. During the 215 days (~7 months) that the traditional approaches were used, the average rate of interest was 0.3 (0.2) women/day, whereas the 26 days of Facebook advertisements resulted in an average rate of interest of 2.8 (1.7) women/day. Facebook advertisements cost Can $506.91 with a cost per eligible participant of Cad $20.28. In comparison, the traditional approaches cost Cad $1087, with approximately Cad $24.15 per eligible participant. Demographic characteristics of women were similar between the 2 recruitment methods except that women recruited using Facebook were significantly earlier in their pregnancy than those recruited using traditional approaches (*P*<.03).

**Conclusions:**

Paid Facebook advertisements hold promise as a platform for reaching pregnant women. The relative ease of placing an advertisement, the comparable cost per participant recruited, and the dramatically improved recruitment rates in comparison with traditional approaches highlight the importance of combining novel and traditional recruitment approaches to recruit women for pregnancy-related studies.

**Trial Registration:**

ClinicalTrials.gov NCT02711644; https://clinicaltrials.gov/ct2/show/NCT02711644 (Archived by WebCite at http://www.webcitation.org/6kKpagpMk)

## Introduction

The recruitment portion of human research studies is often expensive and resource intensive. Finding interested and eligible participants can be challenging and can take longer than expected. Extending the recruitment period can negatively affect time-sensitive study funding, delay data collection and analyses, and ultimately delay the release of evidence necessary to change practice. For prenatal studies, an additional challenge is to find women at an appropriate gestational age.

Traditional recruitment approaches for prenatal studies include printed posters and brochures, radio, television, and newspaper advertisements, word of mouth, and approaching pregnant women in clinics [1]. Placing materials and/or study staff within family physician offices, public health centers, and community centers requires the investigators to establish working relationships with other individuals or agencies, which also takes time. Several investigators have highlighted the potential of supplementing traditional recruitment approaches with social media–based approaches to improve effectiveness of recruitment for clinical studies involving pregnant women [2,3]. Social media, including Facebook, is more than a way to connect with friends and has evolved into a platform for sharing information [3] as well as an effective location for crowdfunding for various causes [4]. With an average of 1.09 billion users daily [5], Facebook is the social media site that individuals engage in most often [6]. The greatest proportion of users are women between the ages of 18 and 49 years [6], which highlights its potential to recruit participants for prenatal studies. Women are likely to engage on Facebook by “sharing statuses” and “liking” posts, which may allow information to be quickly passed along to Facebook friends [4,7].

Edmonton, Alberta, has been noted previously to be a difficult center in which to access and recruit pregnant women for research [1], and Facebook advertisements have proved useful for other studies recruiting participants from “hard to reach” populations. For example, parents of 13- to 17-year-olds [8], immigrants with language barriers [9], individuals at high risk for human immunodeficiency virus infection [10], adolescents [11,12], and young adult veterans [13] have all been successfully recruited using this approach. Advertising to potential participants via Facebook is novel within maternal health research. One study found social media effective for recruiting women before conception [3], while 3 other studies used paid Facebook advertisements to successfully recruit participants for preconception [14] or prenatal studies [15,16]. Each of these pregnancy-related studies involved Internet-based or telephone interventions; thus, there is little known about the potential for Facebook to be used to recruit pregnant women to a randomized controlled trial (RCT) involving face-to-face clinic visits.

The overall objective of the RCT was to examine the efficacy of supportive prenatal counseling versus standard prenatal care in promoting appropriate weight gain and dietary intake among pregnant women. The recruitment goal was 70 healthy pregnant women between 8 and 20 weeks’ gestation, living in the greater Edmonton area. The purpose of this paper was to evaluate the effectiveness of paid advertisements on Facebook as a platform for recruiting pregnant women to this RCT in comparison with traditional recruitment approaches.

## Methods

### Recruitment

**Figure 1:**
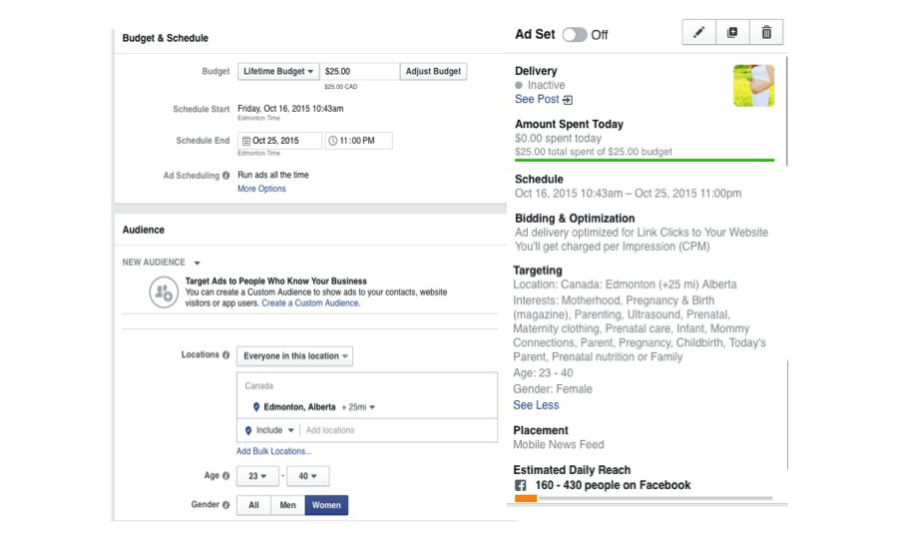
A screenshot that shows the set-up of a paid Facebook advertisement.

**Figure 2:**
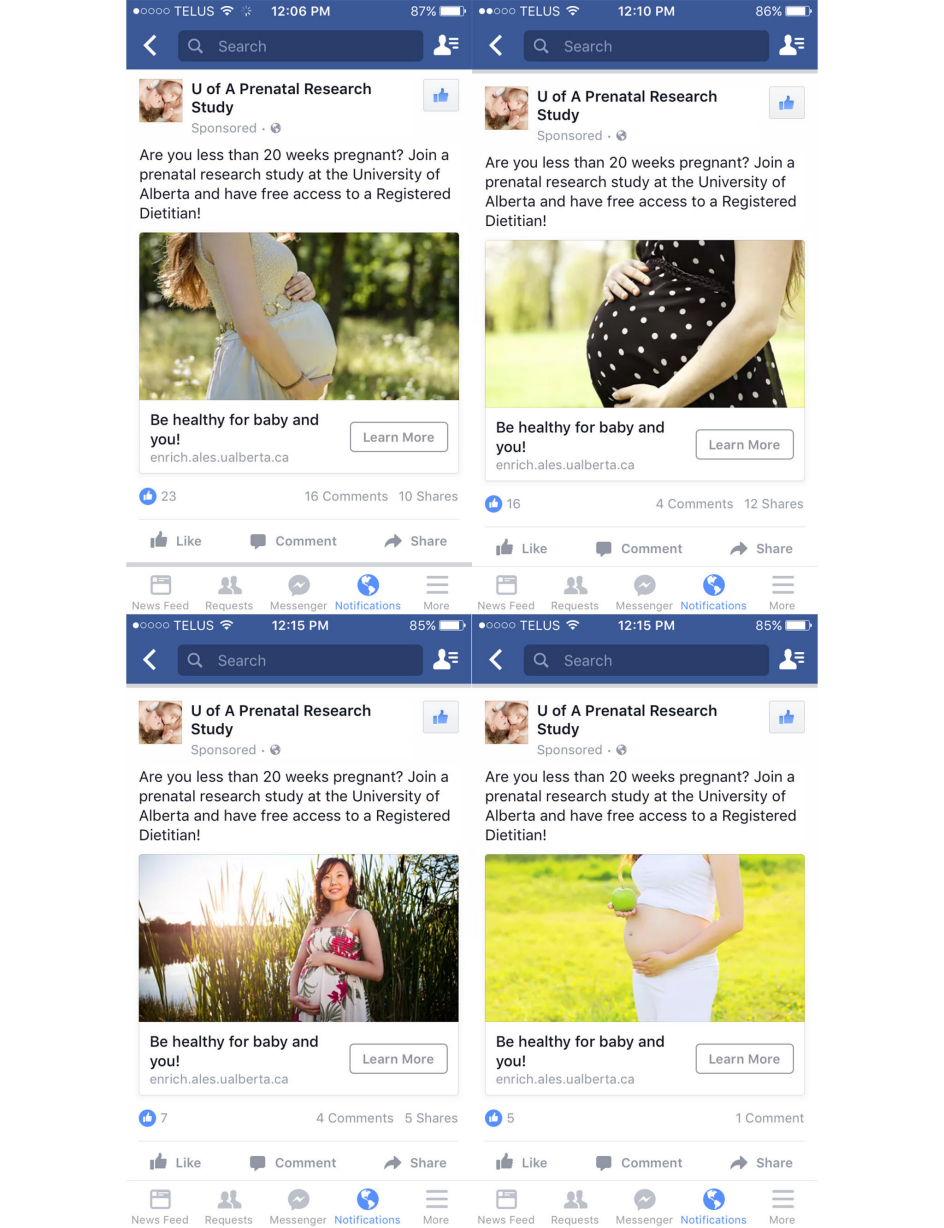
A screenshot that shows the paid Facebook advertisements on a mobile phone.

Recruitment for the RCT using traditional approaches, including printed posters and brochures, word of mouth, newspaper advertisements, local television news health report, booths at mommy and baby fairs, and advertisements in physicians’ offices, started in July 2015. A Facebook account was created to distribute paid advertisements that ran intermittently from October 6 to December 1, 2015, for 26 nonconsecutive days. Advertisements were targeted to Facebook users based on the following criteria: female, 23-40 years of age, living in the Edmonton + 25-mile radius geographic area, and “Interests” related to pregnancy. The “Interests” used were Childbirth, Infant, Maternity clothing, Mommy connections, Parent, Prenatal, Prenatal care, Prenatal development, Motherhood, Today’s Parent, Ultrasound, Prenatal nutrition, Family, and Parenting (Figure 1). Advertisements were managed with a set lifetime budget and Facebook performed the automatic bidding. Advertisements were optimized to get the most number of clicks to the study website at the lowest cost, with fees charged per impression. All Facebook advertisements used the same wording with the headline “Be healthy for baby and you!” and the description text: “Are you less than 20 weeks pregnant? Join a prenatal research study at the University of Alberta and have free access to a Registered Dietitian!”; multiple photographs were tested (Figure 2). Interested women were prompted to click on the “Learn More” button, which took them to the study website [17] where details about the study were provided, including the study contact information. Potential participants were encouraged to contact study staff, after which an appointment was set for screening to determine participant eligibility. Informed consent was provided by the participant in writing before the start of data collection at the baseline visit.

Performance of Facebook advertisements was monitored regularly and optimized in real time using Facebook Ads Manager. Following the trial of multiple paid Facebook advertisement campaigns, the advertisement performance was assessed by examining number of clicks and the amount of engagement. Engagement can be defined as interactions with the study advertisement such as likes, comments, or shares. Funds were reallocated to the Facebook advertisements with the highest number of clicks and highest rate of engagement. Advertisement performance was most affected by the location of the advertisement and the “Interests” specified. Advertisements located on the mobile newsfeed performed better than those on desktop newsfeed; therefore, desktop advertisements were turned off after 9 days. The “Interests” *Prenatal nutrition*, *Family*, and *Parenting* were removed after 9 days owing to poor performance determined through analytics produced from a free trial of AdEspresso, a platform that provides additional statistics to optimize paid Facebook advertisements [18].

Costs associated with each recruitment approach were recorded and used to calculate the cost to the study to recruit eligible participants. The time required by staff to support each of these approaches was not captured and therefore not included in the cost calculations. The local television news report (ie, news reporter, film crew, television airing of segment) was donated in kind, and therefore the costs associated with it were also not included in determination of costs.

### Statistical Analysis

Advertising performance statistics were collected from information provided by Facebook Ads Manager. Demographic information was self-reported by participants who were eligible and had consented to study participation. These data were collected through the Research Electronic Data Capture (REDCap) Consortium member site housed at the University of Alberta [19]. We used *t* tests or Pearson chi-square tests, as appropriate, to investigate differences between groups recruited on Facebook and through traditional means. A *P* value of .05 was considered statistically significant, and all data were analyzed using Stata 14.1 (StataCorp. 2015. Stata Statistical Software: Release 14. College Station, TX, USA: StataCorp LP).

Ethics approval for the RCT was obtained from the Health Research Ethics Board–Health Panel at the University of Alberta (study ID number: Pro00054360). This study is registered at ClinicalTrials.gov (ID: NCT02711644).

## Results

### Recruitment

**Figure 3:**
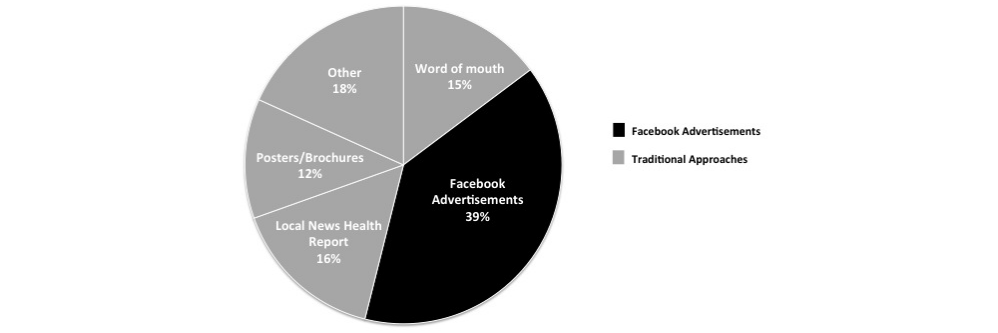
Proportion of interested women for the randomized control trial using Traditional approaches and paid Facebook Advertisements (n=115). The Traditional Approaches include methods listed in addition to ‘Other’ methods that consist of: Online classified advertisements, Doctor referrals, blog posts, email newsletters, newspaper advertisements, mommy markets and online mom connection groups.

A total of 11 women (11/126, 8.7%) did not indicate how they heard about the RCT and were excluded from further analyses. Of the 115 women included in analysis, 39.1% (n=45) of the women who contacted study staff heard about the study through Facebook, whereas 60.9% (n=70) of them heard about it through traditional approaches (Figure 3). Paid Facebook advertisements were received by 44,439 people on their Facebook newsfeed and 1001 Facebook users advanced to the study website, resulting in a click-through rate of 2.3% to the study website. Because of the nature of traditional approaches, the reach of recruitment efforts is not known.

During the 215 days (~7 months) that the traditional approaches were used, the average rate of interest was 0.3 (0.2) women/day. The local television news report resulted in 18 women contacting the study and was the most successful component of the traditional approach. When the local television news report is excluded, the remaining traditional approaches had an average interest rate of 0.2 (0.1) women/day. In comparison, the 26 days of Facebook advertisements resulted in an average interest rate of 2.8 (1.7) women/day. The traditional approaches alone (ie, before launching Facebook advertisements) had an overall interest rate of 8.7 women/month. Adding Facebook advertisements to traditional recruitment approaches increased the overall interest rate to 29.7 women/month. Of note, the Facebook advertisements ran for 26 nonconsecutive days to avoid advertisement fatigue. The maximum number of consecutive days the advertisement was displayed was 8. Advertisements were stopped on day 8 as the performance statistics dropped on days 6-8.

The 45 women who contacted study staff in response to the Facebook advertisement resulted in 40 women who were screened and 25 who were eligible and agreed to participate in the study (55.6% of interested). This resulted in a recruitment rate of 0.96 eligible participants/day. Of the 70 women who expressed interest through traditional approaches, 64 were screened and 45 were eligible and agreed to participate (64.2% of interested), resulting in a recruitment rate of 0.21 eligible participants/day. The calculated (hypothetical) amount of time needed to recruit 70 women using only traditional approaches is 334 days and could be shortened to 73 days using Facebook advertisements.

Facebook advertisements cost Cad $506.91, with a cost of Cad $0.28 per click. Other forms of engagement with the Facebook advertisements included 55 likes, 24 comments, and 28 shares. Most of the comments consisted of the names of Facebook friends who would be notified of the tag. A few comments required response from the study team as they asked questions regarding the eligibility criteria. Facebook advertisements had a cost per eligible participant of Cad $20.28. In comparison, the traditional approaches cost Cad $1087 for all methods combined (ie, printing, media advertisements, mileage to deliver brochures to different venues, participation at mommy and baby fairs). Traditional approaches cost approximately Cad $24.15 per eligible participant.

### Participant Characteristics Relative to Their Method of Recruitment

Women recruited to the randomized controlled trial using traditional approaches and paid Facebook advertisements (n=115). The traditional approaches include methods listed in addition to other methods that consist of online classified advertisements, doctor referrals, blog posts, email newsletters, newspaper advertisements, mommy fairs, and online mom connection groups.

**Table 1:** Characteristics of women recruited using Facebook advertisements versus traditional approaches.

Characteristic	Recruitment method		*P* value
	Facebook advertisements (n=25)	Traditional approaches (n=45)	
Age, years, mean (SD)	35 (4.6)	34 (4.5)	.42
Gestational age, weeks, mean (SD)	12.6 (3.7)	14.7 (3.8)	.03
Prepregnancy BMI^a^, kg/m^2^, mean (SD)	26.4 (5.9)	24.8 (4.9)	.25
**Birthplace, n (%)**			
Born in Canada	22 (88)	36 (80)	.40
Not born in Canada	3 (12)	9 (20)	
**Marital status, n (%)**			
Single	1 (4)	2 (4)	.92
Married or common-law	24 (96)	43 (96)	
**Education, n (%)**			
Less than bachelor’s degree	6 (24)	17 (38)	.24
Bachelor’s degree or higher	19 (76)	28 (62)	
**Household income (Cad $), n (%)**			
< $70,000	2 (8)	8 (18)	.26
> $70,000	23 (92)	37 (82)	
**Employment hours, n (%)**			
Part time	6 (24)	11 (24)	.97
Full time	19 (76)	34 (76)	
**Parity, n (%)**			
One	7 (28)	18 (40)	.17
Two or more	3 (12)	2 (4)	

^a^BMI: body mass index.

## Discussion

### Principal Findings

This study highlights the improved time efficiency achieved by coupling Facebook advertisements with traditional approaches for recruitment of pregnant women to a research study. After a 3-month period of traditional recruitment, we decided to test Facebook recruiting. Along with traditional recruiting, we used Facebook for 26 nonconsecutive days. Had we relied solely on traditional methods of finding interested women, it would have taken close to 1 year to reach our recruitment target, and this was shortened to less than 6 months by adding Facebook advertisements as a recruitment method. This improvement in recruitment rates of pregnant women is similar to that reported by a study recruiting women before conception. Shere et al found that social media–based recruitment resulted in a 12-fold higher rate of recruits per month [3]. It was suggested that the improved recruitment rates could reflect the fact that these types of social media are “active” because the platforms find women based on their previous Web searches related to the research topic, whereas traditional recruitment approaches may be considered “passive” because women likely come across the research opportunity by chance [3]. Our study adds to the evidence indicating that social media holds promise for informing this population about research and recruiting them to participate. Mass media as a whole shows value in recruiting for prenatal research studies, as more than half (54.8%; 63/115) of the women who expressed interest in the study became aware through Facebook advertisements or the local news station health report. Ultimately, social media and other emerging means of mass communication hold promise as means to complement traditional strategies used for recruiting participants because they can reach a large number of people in a short amount of time.

On the surface, the absolute costs of the 2 recruitment methods used in this study were comparable (Cad $24.15/eligible participant for traditional approaches and $20.28/eligible participant for Facebook advertisements). Neither of these costs included those incurred by study staff. Staff or trainee time, mileage, and other related costs can be difficult to measure. The time required to generate and post Facebook advertisements is typically less than that needed to pick up and distribute posters and brochures to multiple sites throughout a large city. One of our traditional approaches, the television health report, was donated “in kind” and involved a local news anchor along with a 1-person film crew. This would have been very costly had the study not been seen as a valuable news item. The free television health report is considered a traditional approach to recruitment. However, if its contribution to the traditional recruitment rate is removed, the cost per eligible participant for traditional approaches increases to Cad $33.97. Future studies should track resource investments, such as staff time, more thoroughly to better understand the relative savings or costs of social media compared with traditional recruitment approaches.

To our knowledge, no additional studies have been published within the last 2 years examining paid Facebook advertisements as a recruitment tool for prenatal studies. Changes in reach, access, and usage of Facebook over the 5 years that these studies and our study span make it difficult to fairly compare results between studies. Our Facebook click-through ratio was better than the 0.08% reported in the study by Arcia [15] but less than the 5.3% on the most successful newsfeed advertisement reported by Harris et al [14]. Arcia was recruiting nulliparous women at less than 20 weeks’ gestation for a Web-based survey [15], whereas Harris et al aimed to recruit young women for completion of a questionnaire on contraception methods [14]. However, because both of these studies recruited between 2011 and 2013, the 2- to 5-year difference makes it difficult to compare them with our study in a fair way. It is possible that changes in the number or characteristics of people who regularly use Facebook along with changes in the features that Facebook provides to paid advertisers could contribute to the observed differences in click-through ratios. Social media certainly has excellent potential to aid recruitment efforts, although the magnitude of change over time remains difficult to quantify.

Our cost per advertisement click was slightly less than those reported by others. In several other studies, cost/click ranged from Cad $0.39/click [20] to Cad $0.45/click [12] and Cad $0.63/click [15]. Differences in the cost per click may vary for different demographic groups. Unique aspects of the target population need to be considered when formulating Facebook advertisements and when choosing topics of interest that help to identify and target these advertisements. Cost per click may continue to vary as Facebook and other social media platforms are modified, potential participants gain experience with using these platforms, or the platforms gain or lose active users. Relative to Facebook and other platforms that promote information sharing, one advertisement may reach more people than originally targeted [21] through features such as a “like, share, or comment.” This can positively result in a more extensive social network of people being notified of a study than what was originally selected [22].

This study suggests that Facebook is a promising platform for reaching and recruiting pregnant women to a research study. Facebook advertisements can be more targeted than many of the traditional approaches because it is possible to define specific demographic and geographic characteristics, and information can be directed to those who search terms on the Web that align with the “Interests” specified by the researcher. Specifying “Interests” related to pregnancy likely enhanced the effectiveness of our advertisements because pregnancy is often a life event that is shared on social media platforms, and Facebook can display the advertisement to users based on their recent activity on the Web. Our RCT is the first prenatal study to describe the “Interests” used in their advertisements. This could be advantageous by reducing the time needed to identify interested participants for a research study. Because most women use the Web to seek health-related information related to pregnancy, Facebook also holds potential as a useful way to distribute public health messages by using “Interests” to target this population [21,23]. An important consideration for Facebook advertisements is striking the balance between advertisement specificity and reach [8]. Targeting an audience with certain characteristics captured by Facebook could allow an advertisement to be cost-effective to reach the target population, but there is a possibility that some individuals will be missed. With approximately 27% of pregnancies being unplanned in Canada, it is probable that Facebook will miss these potential recruits [24]. Individuals who do not use social media or do not have Web-based activity related to pregnancy would also fail to alert the algorithms and may be missed. Therefore, utilizing the combination of traditional recruitment approaches and social media–based approaches is ideal to avoid this selection bias [25].

Previous studies have noted a concern that recruitment through Web-based methods may result in a nonrepresentative population [26]. We found that both approaches recruited women with similar demographic characteristics. Although not significant, a trend may exist of higher education and income within the Facebook group. Our study may not have been powered to detect difference in these populations. Two Australian studies also recruited a fairly nationally representative sample of females within the ages of 18-25 years using Facebook advertisements [11,14]. In our study, the one difference between women recruited through Facebook and women recruited through traditional approaches was that those recruited through Facebook were at an earlier gestational age. This was a promising finding because it is often a challenge to recruit women early in pregnancy [27,28]. Multiple investigators have noted the need for this in prenatal research [25,29-31], and our findings suggest that Facebook advertisements could prove helpful in this regard. Similarly, Richardson et al [29] found that their Internet-based advertisements recruited women significantly earlier in pregnancy compared with other methods.

### Limitations

There are several limitations to this study. Our research team had little experience with Facebook advertisements before starting the study. The advertisements might have been more impactful or cost-effective if those formulating them had more experience developing, testing, and monitoring social media platforms and related statistics. Another limitation was that we could not determine whether women saw the advertisement on their personal Facebook page or if it came to them through a friend who saw it on Facebook. In our study, “word of mouth” was labelled as a traditional approach; however, the original study awareness may have originated from Facebook advertisements. This would underestimate the effectiveness of Facebook advertisements as a recruitment tool.

### Conclusions

With ever-changing technology, researchers must stay current and utilize innovative approaches to find interested study participants. The ease of placing an advertisement on Facebook, the comparable cost per participant recruited, and the dramatically improved recruitment rates when Facebook advertisements were added to traditional approaches highlight the importance of combining novel and traditional recruitment techniques to efficiently recruit women to pregnancy-related research studies, even in geographic areas where recruitment is difficult [1]. Future research should identify the best ways to target pregnant women using Facebook advertisements and other forms of social media to capture a broader range of the resources needed and costs associated with different approaches to recruiting these women.

## Abbreviations

RCT: randomized controlled trial
